# Facing herbivory on the climb up: Lost opportunities as the main cost of herbivory in the wild yam *Dioscorea praehensilis*

**DOI:** 10.1002/ece3.3066

**Published:** 2017-07-11

**Authors:** Bruno Di Giusto, Edmond Dounias, Doyle B. McKey

**Affiliations:** ^1^ International College Ming Chuan University Taipei Taiwan; ^2^ CEFE UMR 5175 CNRS – Université de Montpellier – Université Paul Valéry Montpellier – EPHE Montpellier Cedex 5 France; ^3^ Institut de Recherche pour le Developpement (IRD) Montpellier France; ^4^ Institut Universitaire de France Paris France

**Keywords:** monocotyledon, perennial, plant architecture, plant/herbivore interactions, resource storage

## Abstract

Plants with simple architecture and strong constraints on their growth may offer critical insights into how growth strategies affect the tolerance of plants to herbivory. Although *Dioscorea praehensilis,* a wild yam of African forests, is perennial, both aerial apparatus and tuber are annually renewed. Each year, the tuber produces a single stem that climbs from the ground to the forest canopy. This stem bears no leaves and no branches until it reaches optimal light conditions. Once in the canopy, the plant's production fuels the filling of a new tuber before the plant dies back to the ground. We hypothesized that if deprived of ant defense, the leafless growth phase is a vulnerable part of the cycle, during which a small amount of herbivory entails a high cost in terms of loss of opportunity. We compared the growth of stems bearing ants or not as well as of intact stems and stems subjected to simulated or natural herbivory. Ants reduce herbivory; herbivory delays arrival to the canopy and shortens the season of production. Artificially prolonging the stem growth to the canopy increased plant mortality in the following year and, in surviving plants, reduced the stem diameter and likely the underground reserves produced. Tuber size is a key variable in plant performance as it affects both the size of the aerial apparatus and the duration of its single season of production. Aerial apparatus and tuber are thus locked into a cycle of reciprocal annual renewal. Costs due to loss of opportunity may play a major role in plant tolerance to herbivory, especially when architectural constraints interact with ecological conditions to shape the plant's growth strategy.

## 1 INTRODUCTION

Although the relationships between growth and defense are a focal point in many theories of plant defense, the diversity of plant growth strategies is not yet well integrated into this body of theory. Much interest has centered on how allocation of resources to defense affects the plant's intrinsic potential for growth, and on how growth rate affects tolerance to herbivory (Herms & Mattson, 1992; Strauss & Agrawal, 1999). Moreover, research has focused on how the plant organizes its growth in space and time, and on the consequences of this growth strategy in limiting or responding to herbivory (Boege & Marquis, 2005; Marquis, 1996; Stowe, Marquis, Hochwender, & Simms, 2000; Strauss & Agrawal, 1999). Plants vary greatly in how, where and when resources for growth are stored, mobilized and deployed (Chapin, Schulze, & Mooney, 1990). The cost of herbivory thus varies, not only among different plant parts (Marquis, 1992a) but also for the same plant part at different times (Marquis, 1992b; Marquis, Newell, & Villegas, 1997; Strauss & Agrawal, 1999; Tiffin, 2002). Furthermore, the number and distribution of meristems greatly influence the tolerance of a plant to herbivory (Marquis, 1996; Stowe et al., 2000; Tiffin, 2000).

Monocotyledonous plants differ from most other vascular plants in some basic features of anatomy and development (Tomlinson, 1995), such as the absence of a vascular cambium and thus of secondary growth. This trait constrains growth strategies, tolerance to herbivory and the evolution of defenses (Tomlinson, 1980). First, the absence of secondary vascular cambia severely limits the number of meristems and the repair of damaged tissues (Tomlinson, 1980). Because of the limitation of branching possibilities, many monocots are highly vulnerable to herbivores that destroy meristems. As an extreme example, palms incapable of branching due to their single apical meristem are completely intolerant of such herbivory (De Stevens & Putz, 1985; Tomlinson, 1980). Secondly, because monocots usually develop solely by primary thickening, the construction of any sizable stem requires a phase of initial thickening termed « establishment growth » (Holttum, 1955; Tomlinson, 1980; Tomlinson & Esler, 1973). In this study, we define as establishment growth the slow phase of initial stem thickening and as exploratory growth the subsequent phase of rapid height elongation.

We are unaware of any study that examines how all these traits combine to affect the tolerance of the plant to herbivory at different stages of development, and to shape its adaptations against herbivores. We undertook such a study on a monocotyledonous vine of African seasonal rainforests, the wild yam *Dioscorea praehensilis* Benth. (Dioscoreaceae). We hypothesized that the growth strategy of this plant, shaped by ecological and developmental constraints, should lead to a weak point in the cycle, where a small amount of herbivory could entail a very high cost.

*Dioscorea praehensilis* is a perennial geophyte whose aerial apparatus and underground storage organ are both annually renewed. During the driest part of the year, the plant consists of an underground tuber, whose stored reserves fuel the growth of a single stem to the canopy when growth conditions become favorable. This stem usually does not branch and bears no foliage leaves, only reduced leaves termed cataphylls, until it reaches sunlit conditions. The plant then deploys a crown of leaves covering the canopy vegetation, sometimes situated at 30 m or more above the ground. Production by these leaves over several months then fuels the filling of a new tuber, replacing the plant's exhausted underground reserves. Finally, after the end of the long rainy season, the entire aerial system dies. This annual cycle of renewal of the vine's crown and of the stored underground reserves is the central feature of the morphology, physiology, and phenology of the plant.

A reduction in the efficiency of this cycle of reciprocal renewal could be costly to the plant. We hypothesized that rapid height growth during the climb to the canopy should be crucial to the plant's success, because photosynthetic production starts only after reaching the canopy and because the plant's aerial system has a limited lifespan. Growth in height is maximized by the production of long internodes and by the absence of branching. The climbing phase to the canopy thus appears to be the most vulnerable to herbivory. Damage to the single apical meristem necessitates the activation of a lateral bud to take over vertical growth. To produce such a ramification could delay reaching the canopy, reduce the length of the single season available for production, and result in loss of opportunity for photosynthesis by the plant's entire crown. Should herbivory occur during this phase, the high cost in terms of lost opportunities should have long‐term consequences. First, a smaller window of photosynthetic production could reduce the amount of reserves stored in the tuber. Second, this lowered storage could in turn reduce both the rate of growth to the canopy and the photosynthetic capacity (both the duration of the productive season and the photosynthetic surface that can be deployed) of the following year's shoot. Once the cycle is weakened, the plant could be caught in a downhill spiral.

The cost of herbivory during this phase of growth has two components, cost of lost material and cost of lost time. Because construction cost of the apical meristem is probably small relative to the amount of reserves stored in the tuber, the material cost of replacing the small amount of meristem tissue destroyed by herbivores is likely to be small. In contrast, the cost of meristem herbivory in terms of lost time for photosynthetic activity and replenishment of reserves is likely to be considerable and long lasting. Herbivory that delays arrival to the canopy, where the plant can accumulate enough reserves to repeat the trip in the following year, could greatly reduce the plant's fitness. The height of the canopy and the amount of accumulated reserves in the tuber should thus be crucial variables affecting the overall success of the strategy. Failure to replenish the tuber reserves should result in increased mortality, decreased growth performance, or both, thus extending and even amplifying the effects of herbivory over time.

One of the traits of *D. praehensilis* supporting the hypothesis of a high cost of herbivory during the phase of rapid growth through the understory is the presence of both direct (saponins) and indirect antiherbivore defenses (biotic defense via extrafloral nectaries). The latter type of defense appears to be specific to this growth phase. The extrafloral nectaries, solely active on cataphylls near the apical meristem, attract more than 30 species of opportunistic ants. The effectiveness of this biotic defense varies from year to year due to the opportunistic nature of the protection mutualism and the efficiency of the anti‐ant defenses of the principal herbivore in our study sites, *Lilioceris latipennis* Clark (Chrysomelidae: Criocerinae) (Di Giusto, Anstett, Dounias, & McKey, 2001). Indeed, larvae of this beetle subvert the plant's chemical defenses, incorporating them into a fecal shield in their own defense against ants (Di Giusto et al., 2001). The fact that a single specialist phytophagous insect strongly predominates among the herbivores we observed on the plant is consistent with well‐developed defenses, likely driven by a high cost to the plant were herbivory to occur.

We studied the effect of herbivory on the time required by growing stems to reach the canopy. We compared the growth of intact stems with that of stems subjected to natural or simulated herbivory. Simulation of herbivory allowed us to control sources of variation (in the timing, location, and severity of attack) in actual attacks. We also simulated the effects of a higher canopy by artificially prolonging the time required by stems to reach the canopy where they could begin to replenish stored reserves. Our observations and experiments were designed to address the following questions: (1) Does herbivory affect the time required for the stem apex to reach the canopy? (2) If so, do ants minimize the impacts of herbivory on the plant's growth? (3) Is the onset of production delayed and the productive period shortened after herbivory? (4) What is the relative importance of herbivory for establishment versus exploratory growth? (5) Does the quantity of reserves stored in the tuber affect the time required by the stem to reach the canopy? (6) How would a delay in reaching the canopy affect the replenishment of tuber reserves, probability of survival, and growth of surviving individuals in the following year?

## 2 MATERIALS AND METHODS

Fieldwork was conducted in a 25‐year‐old secondary forest near the village of Kouedjina, Eastern Province, Cameroon (3°55′N, 13°45′E). Vegetation in this region is typically semideciduous forest (Letouzey, 1985). Annual rainfall is 1760 mm (average over 6 years from 1988 to 1993; data from the weather station of the logging company Société d'Exploitation Forestière des Bois du Cameroun (SEBC) located about 30 km from the study site). The climate is characterized by four distinct seasons; fieldwork was conducted between January and June, that is, from shortly before to shortly after the short rainy season (March to May) in 1999, 2000, and 2001.

**Figure 1:**
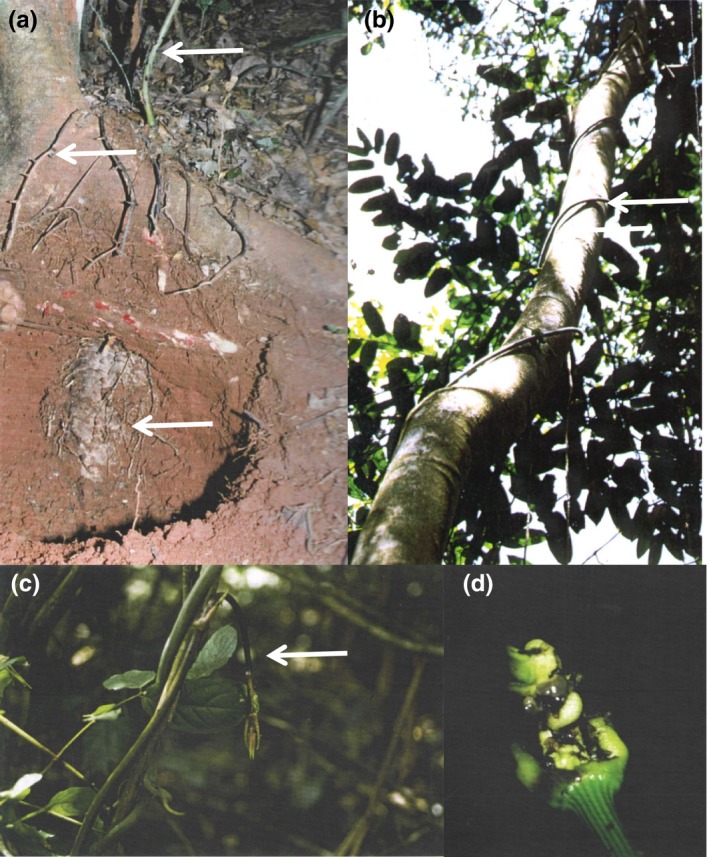
Description of *Dioscorea praehensilis*. (a) The tuber usually produces only one stem. The upper top part of the tuber (tuber head) is perennial and protected from digging animals by thorny roots; the bulk of the tuber is annually renewed (gray mass in the photograph). The arrows show from top to bottom the stem, the thorny root, and the tuber. (b) The stem of *D. praehensilis* bears neither leaves nor branches on its way to the canopy. (c) Growing intact shoot apex before herbivory. (d) Larvae of *Lilioceris latipennis* have caused the destruction of about 20 cm length of the growing shoot apex. The shiny gray objects are larvae of *L. latipennis*, each covered by a glistening fecal shield

*Dioscorea praehensilis* grows in the forested zones of western and central Africa (Burkill, 1985; Hladik, Bahuchet, Ducatillon, & Hladik, 1984). The growth cycle of this geophyte has been described by McKey, Di Giusto, Pascal, Elias, and Dounias (1998) and Di Giusto et al. (2001). The tuber head is perennial, but the bulk of the tuber (up to 28 kg fresh weight) is used up and replaced each year (B. Di Giusto and E. Dounias, unpublished data) (Figure 1a). Each year, to renew the plant's photosynthetic apparatus, reserves stored in an underground tuber fuel the growth of a single stem to the canopy. The plant is light‐demanding, and its crown of leaves is usually found in fully exposed canopy vegetation. *Dioscorea praehensilis* is not found in primary forest with very high canopies, where it is replaced by other yam species with perennial tubers and stems that are renewed at biennial or longer intervals, such as *D. mangenotiana* (Hladik et al., 1984).

At the end of the dry season (January–February), the plant consists solely of the tuber and some buds located at the perennial uppermost head of the tuber. In all 3 years of study, the first stems to emerge from the ground were observed in the first 2 weeks of January. Stem emergences peaked between 15 February and 15 March. Most plants had reached the canopy by the end of April, although a few stems were still emerging at this period.

During establishment growth, stems are rigid and self‐supporting for the first 1.0–1.5 m of their length, vertical growth is slow, and internodes produced are short. This establishment growth provides the base required for exploratory growth. The latter is characterized by rapid vertical growth (mean 22.4 cm/day in the absence of herbivory; see below), less robust viny stems, long internodes, and the use of the surrounding vegetation to climb to the canopy (Figure 1b,c). In our study site, the canopy was situated at about 12–15 m. Plants required on average more than 2.5 months to reach the canopy. Once the plant's cohort of leaves has been put in place (late April to early May), the flow of energy is reversed to replenish a new tuber. Flowering and fruiting occur in July and August, after which the aerial shoot gradually withers (over a period of 2 months) and dies by the end of October (B. Di Giusto and E. Dounias, unpublished data).

During its growth through the understory, the apex is frequently attacked by phytophagous insects (Di Giusto et al., 2001). These include grasshoppers (Acrididae) and lepidopteran larvae, but the most frequent herbivore in our study site was the leaf beetle *L. latipennis*. Larvae and adults feed on the tender young stem and cataphylls, often killing the apical meristem (Figure 1d). Adult beetles first appear within 10–15 days after the first rains of the short rainy season (mid‐March), and continue reproductive activity over the entire period of stem emergence. Duration of larval development varies between 1 and 2 weeks from egg hatching to pupation when conditions are favorable (Di Giusto, 2002). An imago can repeatedly lay eggs on the growing area of the same stem or other individuals may add their clutches of eggs to a pre‐existing clutch (B. Di Giusto, unpublished data).

### 2.1 Effect of herbivory on the time required to reach the canopy

To estimate the short‐term cost of herbivory in terms of reduced growth rate of stems of *D. praehensilis*, we compared the lengths of stems produced by different groups of individuals (intact, or subjected to natural or simulated herbivory) over a period of 20 days (22 March–11 April 1999).

We randomly divided 74 plants into three groups, all placed on stakes (2.5 m‐high supports that maintained stem apices at eye level) to facilitate observations. All stems were maintained in near‐vertical position to avoid stems from bending or breaking. A first group of controls (*N* = 25) was naturally patrolled by ants. A second group (“ant‐exclusion”) (*N* = 24) was not patrolled; ants being excluded by a barrier of glue (see Di Giusto et al., 2001 for methods). Finally, a third group (“simulated herbivory”) (*N* = 25) was freely patrolled by ants and subjected to simulated herbivory by clipping the stem beneath the second pair of open (no longer appressed to the stem) cataphylls from the apex. Because we removed a standardized portion of the stem (corresponding to a length of about 10–15 cm from the apex), our evaluation of the effect of herbivory does not take into account the considerable variation in the extent of damage actually inflicted by *L. latipennis* larvae. However, the biggest difference between natural and simulated herbivory was in the time required for the destruction of the shoot apex: immediate cut for the simulated herbivory and gradual destruction over a period of 3–7 days for the natural herbivory (B. Di Giusto, unpublished data).

The ant‐exclusion treatment allowed an estimate of the effect of ant patrols on the probability of herbivory and, when herbivory did occur, its effect on the growth of stems. The simulated herbivory treatment allowed us to increase the total sample size of attacked stems. Standardized treatment also allowed for elimination of extraneous sources of variation that occur when real herbivory intervenes (Tiffin & Inouye, 2000), due to factors such as (in *D. praehensilis*) the type of insect attacking, the precise site of the attack and how much time the insect spent consuming tissues before fully severing the apex.

Because of the difficulty of estimating the exact quantity of tissue consumed by insects, we recorded only two states for the apices, “cut” versus “not cut”. Attacks that cut the apex always entailed destruction or necrosis of the apical meristem and a section, usually about 20 cm in length, of the apical portion of the stem. Our method does not take into account minor attacks that did not kill the apical meristem, but often caused a slight and temporary reduction of growth. In general, the greater the diameter of the stem, the greater the likelihood that the apical meristem will survive an attack (B. Di Giusto, unpublished data).

#### 2.1.1 Global analysis of stem growth over 20 days

We used a General Linear Model (R Core Development Team 2013) to estimate the effects of natural herbivory, simulated herbivory, and the absence of patrolling ants on stem growth. We assessed the effects of various explanatory variables [simulation of herbivory (“cut” vs. “not cut”, number of times the apex was cut by natural herbivory (0 = intact, 1 = cut once, 2 = cut twice), the presence of ants (“ant‐exclusion” vs. “ant presence”), the presence of larvae (“absence” vs. “presence”), cross‐sectional stem area (at 1 m above ground), and initial length of the plant on the total stem length produced in 20 days. The presence of larvae (main source of natural herbivory) at any point during the experiment was checked every other day on all stems. The presence of ants was similarly checked on both control and clipped stems; the absence of ants was confirmed on ant‐excluded stems. The model with the best fit was chosen using the AIC criterion, adjusted R‐square and comparing the different models among themselves using ANOVAs (“Car” package, R Development Core Team, 2013).

#### 2.1.2 Detailed analysis of stem growth

For this analysis, measures of stem length were carried out every 2 days (10 measures) on the control and simulated herbivory groups only. Ant‐excluded plants were not included in this experiment, as frequent measurement of stems risked knocking off some of the numerous *L. latipennis* larvae feeding on those stems. To estimate the time lost in reaching the canopy after real or simulated herbivory, we compared the growth of intact and damaged stems using a repeated‐measure ANOVA. We then used our data over the 20‐day period to estimate by extrapolation the total time lost in reaching the canopy.

This lost time includes three potential components: (1) the time required to activate lateral buds after destruction of the apical meristem, (2) the time required to complete a new round of establishment growth, and (3) the effect of herbivory on the rate of exploratory growth.

First, the number of days required for activation of at least one lateral bud following real or simulated herbivore attack was recorded for control and simulated‐herbivory stems. We considered the activation to be complete when the bud reached 1–1.5 cm in length (resting buds are 1–6 mm in length, depending on diameter of the stem bearing them). We used the number of days necessary for bud activation as dependent variable and the initial length and diameter of the stem, the presence/absence of larvae (at one or more daily observation), and type of damage (real or simulated herbivory) as explanatory variables. We then conducted an ANOVA using a generalized linear model (R Core Team, 2013).

Second, we estimated the time required to complete a new round of establishment growth, which, in the sense of Tomlinson and Zimmermann (1966), includes bud activation, during which the meristem of monocots begins to thicken. In *D. praehensilis*, this phase includes both a slow elongation and a thickening of the stem to provide the rigid base required for the self‐supporting stage. The two phenomena, one peculiar to monocots, the other typical of vines with an initial self‐supporting phase, are difficult to separate.

We used an arbitrary criterion to mark the end of establishment growth: The mean minimum growth rate (13.8 cm/day) of 15 healthy individuals that had begun exploratory growth and bore no trace of herbivory. Among the nine control stems subjected to real herbivory and the 25 stems subjected to simulated herbivory, only a subset reached our arbitrary criterion [real herbivory: *N* = 5, i.e. four control stems plus one simulated‐herbivory stem) and simulated herbivory: *N* = 24]. For each stem of this subset, we recorded the number of days after bud activation required to reach the threshold growth rate. Analysis of establishment growth using a generalized linear model was also conducted, using the same explanatory variables as in the preceding analysis. Repeated damage to the apex, a longer time to bud activation, a prolonged delay in leaving the self‐supporting phase, or a combination of these causes may explain why some of the stems did not reach the threshold minimum growth rate of 13.8 cm/day (marking the end of establishment growth).

During establishment growth, the stem is rigid and self‐supporting. To determine whether this trait was associated with an increase in stem diameter that might compromise its growth in length, we compared the diameter of the stem before attack and the diameter of the new stem produced after destruction of the apical meristem (*N* = 42 sites of herbivory: 25 simulated and 17 natural attacks distributed across control and simulated‐herbivory plants). Diameter was measured at the middle of the internode immediately below the pair of cataphylls bearing the new ramification, and at the middle of the first internode of the new branch. These measures were taken only after complete rigidification (indicated by stiffening) of the internodes.

Finally, to examine whether experimentally damaged stems overcompensated to make up for lost time, or whether, in contrast, damage resulted in a decreased growth rate of the new ramification, we compared the maximal growth rate, after completion of establishment growth, that was reached by intact stems (*N* = 15) and simulated‐herbivory stems (*N* = 22 stems not attacked by herbivores, and receiving only simulated herbivory) at the end of the 20‐day experiment. All control stems presenting either traces of herbivory or the presence of larvae were excluded from this analysis. The maximum growth rate was defined as the highest daily growth rate observed for one of the ten 2‐day intervals for each intact and simulated‐herbivory stem. For simulated‐herbivory stems, these maximum values were attained near the end of the experiment, after establishment growth was completed. We also conducted an analysis using a generalized linear model and the same explanatory variables as in the preceding analyzes (R Development Core Team, 2013).

Using a repeated‐measures ANOVA (nine measures) (SAS 2013), we also compared the slopes (considered along a straight line) between two consecutive length values of exploratory growth between intact (*N* = 15) and simulated‐herbivory stems (*N* = 16; the others failed to exceed our criterion for ending establishment growth). We first compared slopes of growth for each 2‐day interval between treatments, then compared slopes of growth over the 20‐day period for each treatment separately. For this analysis, we considered a type III error as recommended in SAS manual.

### 2.2 Effects of a delay in reaching the canopy

Increased height of the canopy, by necessitating a longer climb, could limit the plant's capacity to renew tuber reserves. To simulate the effects of a higher canopy, from the end of February to the end of April 2000, we artificially prohibited 95 stems of *D. praehensilis* from reaching the canopy. To do this, we regularly removed the apex of the climbing stem from its supports, maintaining it at eye level, and putting it on the surrounding vegetation throughout this period. From May 1 onward, all experimental stems were then allowed to climb to the canopy. Another group of 89 stems, allowed to grow freely, served as controls. Experimental plants were thus subjected both to the cost of delay in reaching the canopy and to the material cost imposed by the continued stem elongation in the understory. Precisely when the stems of the experimental plants reached the canopy is unknown, as the density of the vegetation during the rainy season made it difficult to follow their progress. However, most of these stems, if not all, did eventually reach the canopy, as their dried stems could be seen in the highest parts of the trees in the following dry season. We recorded stem diameter (at 1 m above ground) of the control and experimental stems in April 2000 and April 2001, comparing individual survival (the absence of a stem in 2001 indicated death of the plant) and change in diameter of the stem produced by a plant from 1 year to the next.

The two groups of plants were compared at *T*_0_ using a single‐factor ANOVA to test for homogeneity of groups. Because distributions of initial cross‐sectional area of stems were not homogeneous between the two groups (control stems were slightly but significantly smaller), we compared the proportional difference in cross‐sectional area relative to the year 2000 [*X*_2001_ − *X*_2000_/*X*_2000_], to eliminate the effect of natural variation in stem cross‐sectional area among individuals.

### 2.3 Effect of stem size on the timing of stem emergence and the end of the leafless phase

In 2000, we censused 275 stems on four different dates (12 February, 8 March, 12 April, and 24 April). We recorded on each date the number of stems that had emerged above ground level since the previous census, and whether the shoot had begun to produce true foliage leaves. The diameter of each stem, once rigid, was measured at 1 m above ground. To test whether the stem cross‐sectional area (and thus the quantity of reserves in the tuber that produced it; see supplementary information) affected the timing of stem emergence, we compared the cross‐sectional areas of stems that first emerged during each of the four census intervals, using a Kruskal–Wallis test. We then performed Mann–Whitney tests to compare each pair of groups.

To test whether stem size affected when the plant began to produce foliage leaves, we compared the cross‐sectional areas of the 22 stems that bore foliage leaves by 24 April (18 stems on which leaves were first seen on 12 April, four additional stems on 24 April) with the other plants still in leafless phase at this final census date, using a Mann–Whitney test. Nonparametric tests were chosen for all these comparisons because our data were not normally distributed.

## 3 RESULTS

### 3.1 Effect of herbivory on the time required to reach the canopy

#### 3.1.1 Global analysis of stem growth over 20 days

Larvae of *L. latipennis* were responsible for all the attacks on the growing stems of *D. praehensilis*. Although control stems (seven cut stems of 25) and simulated‐herbivory (three cut stems of 25) stems patrolled by ants suffered less herbivory than ant‐excluded stems (10 cut stems of 24), the difference was not significant (2 × 3 Fisher exact test, two‐tailed, *p* = .06). However, significantly more stems on ant exclusion (15 stems attacked by larvae) bore *L. lilioceris* larvae than control stems (seven stems attacked by larvae) and simulated‐herbivory (five stems attacked by larvae) (2 × 3 Fisher exact test, two‐tailed, *p* = .006).

**Figure 2:**
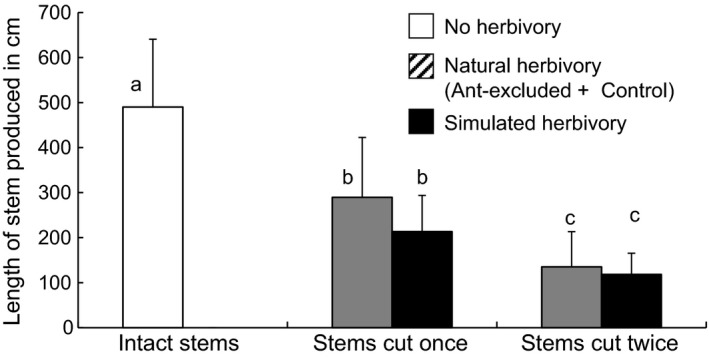
Effects of natural and simulated herbivory on growth over 20 days of stems of *Dioscorea praehensilis*. Control stems (*N* = 25), ant‐exclusion stems (*N* = 24), stems subjected to simulated herbivory (*N* = 25). Histograms and error bars are mean + *SD*. Letters indicate significant differences at α = 0.05 (LSMEAN, Tukey–Kramer test)

The effect of simulated herbivory was significant. Experimentally damaged stems (simulated herbivory) grew much less during the experiment (*N* = 25, mean ± *SD*: 199.4 ± 84.5 cm) than did control stems patrolled by ants (*N* = 25, mean ± *SD*: 448.5 ± 176.0 cm) and stems subjected to ant‐exclusion (*N* = 24, mean ±*SD*: 352.9 ± 194.5 cm) (Table 1). Natural herbivory on ant‐exclusion plants was intermediate in frequency and intensity between simulated herbivory and low natural herbivory on control stems. Herbivory, whether natural (*N* = 18, mean ± *SD*: 245.1 ± 134.6 cm) or simulated (*N* = 25, mean ± *SD*: 199.4 ± 84.5 cm), led to a reduction by 40%–50% in the stem length produced compared to individuals that suffered no herbivory (*N* = 32, mean ± *SD*: 494.8 ± 150.7 cm) (Table 1). The effect of the number of natural herbivory events was also significant, as repeated attack led to further reduction in growth. Stems attacked twice (*N* = 9, mean ± *SD*: 129.5 ± 66.8 cm) grew half as long over the 20 days as stems attacked once (*N* = 33, mean ± *SD*: 238.8 ± 105.3 cm) (Table 1, Figure 2). While simulated herbivory occurred at the beginning of the experiment for all plants, the time at which natural herbivory occurred varied among plants. This explains why the mean length of stems subjected to natural herbivory was both greater and more variable than those of experimentally damaged plants. Furthermore, the presence of larvae also significantly reduced stem growth (Table 1), probably by the infliction of continuous sublethal damage to the growing area.

**Table 1:** Effects of herbivory simulation (cut versus not cut), number of events of natural herbivory (0 = intact, 1 = attacked once, 2 = attacked twice), presence of ants (ant‐excluded or ants present), presence of larvae (present versus absent), cross‐sectional area, and initial length of the plant on lengths of stem produced over 20 days, tested using a generalized linear model with normal error (R Core Team, 2013). The variable « length produced over 20 days » was transformed in log(*x*). Data were tested for normality using the Shapiro‐Wilk test and for homogeneity of variance residuals using Levene's test

	*df*	*F*	*p*
Simulation of herbivory	1	55.67	**4.47 × 10** ^**−10**^
Number of events of natural herbivory	1	97.00	**4.54 × 10** ^**−14**^
Presence of larvae	1	6.89	**.01**
Presence of ants	1	1.02	.32
Cross‐sectional area	1	6.31	**.01**
Initial length	1	4.78	**.03**
Simulation of herbivory* presence of larvae	1	0.10	.75
Simulation of herbivory* initial length	1	0.02	.87
Number of natural herbivory* presence of larvae	1	3.41	.07
Number of natural herbivory* initial length	1	0.32	.57
Presence of larvae* presence of ants	1	0.37	.54
Presence of larvae* initial length	1	5.09	**.03**
Cross‐sectional area* presence of ants	1	6.69	**.01**
Cross‐sectional area* initial length	1	5.37	**.02**

Significant results are in bold.

Stem with large cross‐sectional area had a significantly higher vertical growth rate (Table 1). Similarly, initial stem length was also positively correlated with the rate of stem vertical growth: as stems elongated, their rate of vertical growth increased (Table 1). However, the interaction cross‐sectional area*initial length was also significant, as some stems with large diameter but short initial length had severe growth reduction compared to others with long initial length (Table 1). The presence of larvae on the stem at an early stage of growth increased the risk of repeated attack, as shown by the significant interactions between the presence of larvae*initial length. This could be due to the fact that female *L. latipennis* repeatedly oviposit on the same stems a few days apart (B. Di Giusto, pers. obs.). Finally, the interaction cross‐sectional area*presence of ants was also significant, showing that the effect of cross‐sectional area of a stem on its extension growth was especially important when ants were excluded (Table 1). This could indicate that biotic defense is especially important for young, small plants.

#### 3.1.2 Detailed analysis of stem growth

Of the 25 control stems, we selected 15 whose apices remained intact and that showed no signs of any perturbation of growth (such as the presence of larvae for a period of 1 week or longer, natural mechanical damage, or damage induced by errors of manipulation). After 20 days, the mean extension growth of these stems was 420.4 ± 140.3 cm, that is, a daily growth rate of 22.4 ± 6.17 cm/day. The highest daily growth rate observed for each plant averaged 37.1 ± 17.75 cm/day, with the most rapidly growing plants showing extreme values of up to 74 cm/day. The mean minimal growth rate for all plants was 13.8 ± 4.14 cm/day, with the lowest minimal value observed being 9 cm/day. The mean minimum growth rate of 13.8 cm/day was arbitrarily used as the threshold point marking the end of establishment growth.

If the apical meristem of the shoot was destroyed by herbivores, two lateral meristems, in general the pair situated highest on the plant (usually the distalmost intact node immediately below the site of attack), became active. One of these became dominant, repeating first establishment growth, then exploratory growth.

Our results concerning the three phases in resumption of vertical growth after natural herbivory are presented below. First, after natural herbivory, 2–4 days (*N* = 10, mean ± *SD*: 2.6 ± 0.8 days) were required before lateral buds became active and reached a length of 1–1.5 cm. After simulated herbivory, this delay was 2–8 days (*N* = 25, mean ± *SD*: 4.6 ± 1.5 days). The difference in the time required between the two types of herbivory was significant (*F*_1, 34_ = 14.47, *p* < .0006). An average delay of 4.5 days for activation of a lateral bud is, we believe, a realistic estimate of the time lost for bud activation.

Second, the control (*N* = 4) and simulated‐herbivory (*N* = 1) stems that sustained natural herbivory required 5–8 days (*N* = 5, mean ± *SD*: 6.6 ± 1.1 days) for the new axis to complete a new round of establishment growth. After simulated herbivory, stems required 4–16 days (*N* = 24, mean ± *SD*: 8.5 ± 3.0 days) to accomplish this. None of the explanatory variables tested (type of herbivory, stem cross‐sectional area, initial stem length), nor their interactions, significantly affected the number of days necessary to complete a new round of establishment growth.

After destruction of the apical bud by natural or simulated herbivory, the cross‐sectional stem area of the new ramification (*N* = 42, mean ± *SD*: 68.4 ± 52.3 mm²) was almost double that of the principal stem (mean ± *SD*: 35.3 ± 26.8 mm²) (*N* = 42, paired *t*‐test, *p* < 10^−7^). The cross‐sectional area of the ramification was also positively correlated with the cross‐sectional area of the principal stem measured at 1 m above ground (*R* ^2^ = .37, *p* = .00001).

**Figure 3:**
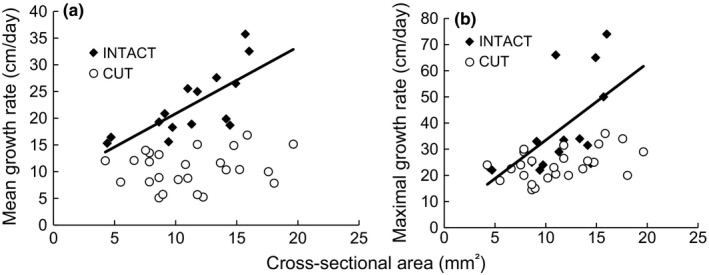
Relation between cross‐sectional area and extension growth rate of the stem. Comparison of growth of intact stems (*N* = 15, black diamonds) and ramifications produced by experimentally damaged stems (*N* = 22, white circles) over 20 days. (a) Mean growth rate (in cm/day) as a function of cross‐sectional area. Cross‐sectional area of the initial axis was significantly correlated with mean growth rate of intact stems (*R* ^2^ = .54, *p* = .002) but not with that of relay axes of experimentally damaged stems. (b) Maximal growth rate (in cm/day) as a function of cross‐sectional area of the initial axis. Cross‐sectional area was significantly correlated with maximal growth rate of intact stems (*R* ^2^ = .36, *p* = .02) but not with that of relay axes of experimentally damaged stems

Third, this analysis included cut stems that sustained either natural or simulated herbivory. Only stems producing a ramification that attained the threshold (13.8 cm/day) chosen to mark the end of establishment growth were considered. By the end of the 20‐day period, the ramifications of the cut stems had not re‐attained the mean rate of extension growth of the intact plants. The mean growth rate of the ramifications (10.6 ± 3.4 cm/day) was only half that of the intact stems (22.4 ± 6.2  m/day) (*F*_1, 36_ = 94.28, *p* = 1.3 × 10^−11^). Furthermore, the larger the cross‐sectional area of the stem (both intact stems and ramifications produced by cut stems), the faster the mean growth rate (*F*_1, 36_ = 11.09, *p* = .002). The interaction cross‐sectional area*herbivory (whether or not stems were cut) was also significant (*F*_1, 36_ = 13.87, *p* < .0007), as only the mean growth rates of the intact stems showed an effect of cross‐sectional area (*N* = 15; *R* ^2^ = .54, *p* = .002; see Figure 3a).

For cut and intact stems taken together, the maximal rate of extension growth was similarly affected by herbivory (natural or simulated) (*F*_1, 36_ = 17.24, *p* = .0002) and by stem cross‐sectional area (*F*_1, 36_ = 11.68, *p* < .002). Ramifications showed maximal growth rates one‐third lower (24.1 ± 5.8 cm/day) than those of intact stems (37.1 ± 17.7 cm/day). The interaction cross‐sectional area*herbivory also had a significant effect on the growth rate (*F*_1, 36_ = 6.97, *p* = .01). Indeed, in contrast to the positive effect of cross‐sectional area on extension growth of intact stems (*N* = 15; *R* ^2^ = .54, *p* = .002), the maximal growth rate of new ramifications did not increase significantly with their initial cross‐sectional area (Figure 3b).

**Figure 4:**
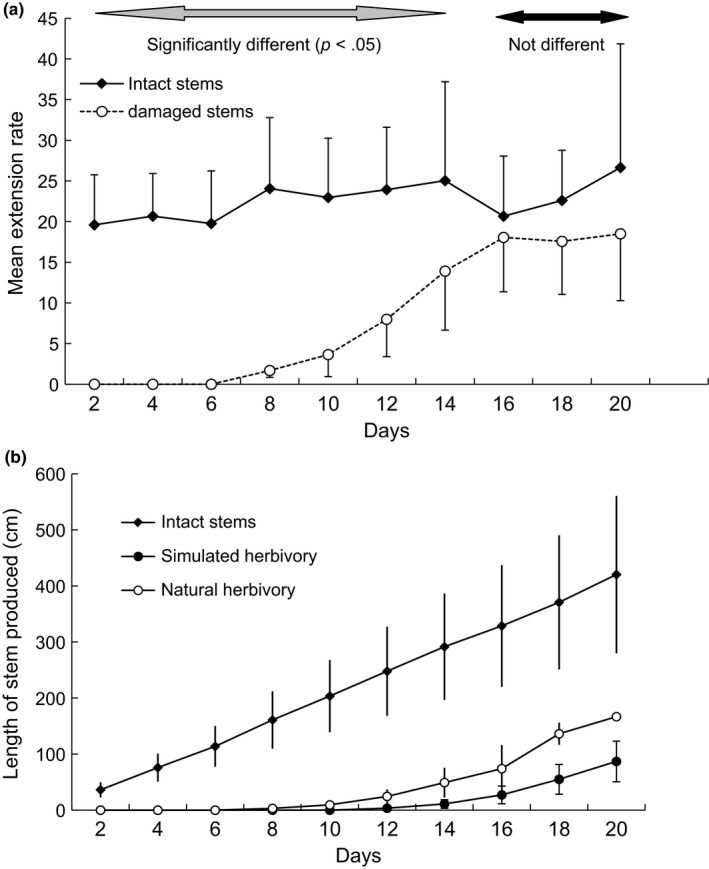
Growth with and without herbivory. (a) Comparison of mean extension rate per 2‐day period for intact stems (*N* = 15) and for experimentally or naturally damaged stems (*N* = 16, stems having attained the threshold point of 13.8 cm/day). Standard deviation bars are oriented upward for intact stems and downward for damaged stems to avoid overlaying. (b) Comparison of length of stem produced over 20 days in cm/day for intact stems (*N* = 15) and for ramifications of stems subjected to simulated (*N* = 21) or natural (*N* = 4) herbivory

Repeated‐measures analysis comparing the slopes of growth of intact and cut stems (how steep a straight line is between two length values at *T*_0_ and *T*_1_) allowed us to examine the persistency of the effect of meristem destruction on the rate of exploratory growth. This analysis showed an effect of cross‐sectional area (*F*_1, 28_ = 23.40, *p* < .0001) and an effect of the interaction cross‐sectional area*treatment (*F*_1, 28_ = 23.40, *p* < .0001). For the other interaction tested, only time*cross‐sectional area*treatment was significant (*F*_1, 224_ = 26.94, *p* < .0001). Comparison of slopes of extension growth rate over time for new ramifications showed that the rates of extension growth during establishment and exploratory growth were significantly different. However, once establishment growth was completed (day 16), there was no significant difference in the slope of growth between intact and cut plants (Figure 4a). Repeated‐measures analysis of the slopes of growth of intact stems showed a strong effect of cross‐sectional area (*F*_1, 13_ = 15.16, *p* = .001), but no time effect. In contrast, the same analysis for damaged stems showed no effect of cross‐sectional area but a strong time effect (*F*_8, 112_ = 4.12, *p* < .0002).

**Figure 5:**
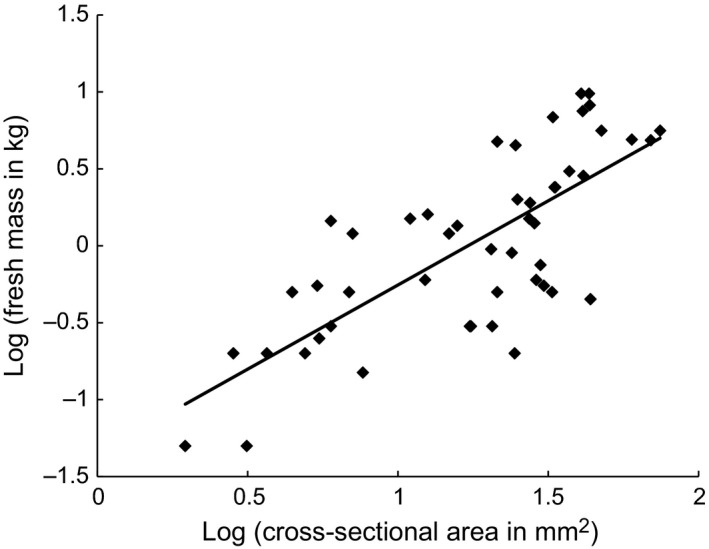
Correlation between cross‐sectional area of stem and fresh mass of tuber of *Dioscorea praehensilis* (*N* = 49 tubers and stems). Data were log transformed. Cross‐sectional area (in mm^2^ (*A* = π*R*²)) of the stem was significantly correlated with the fresh mass (in kg) of the tuber (*R* ^2^ = .55, *p* < 10^−6^). The solid line is the regression (log linear model) of tuber mass on stem cross‐sectional area (*y* = 1.09*x* − 1.35)

Natural or simulated herbivory thus strongly reduced the stem extension growth rate: (1) in the short term (delay due to bud activation and to completion of establishment growth), and (2) in the longer term (reduced rate of exploratory growth of the ramification) (Figure 4a). Following natural or simulated herbivory, individuals of *D. praehensilis* did not compensate by increasing their growth rate. The rate of extension growth of the ramification was strongly dependent on the cross‐sectional surface of the initial stem (and thus, we deduce, on the amount of tuber reserves, but see Supporting information and Figure 5). However, the slope of this relationship was lower than in intact stems. Herbivory thus appeared to reduce the advantage in the extension growth rate conferred by possession of large amounts of tuber reserves.

### 3.2 Effects of a delay in reaching the canopy

Prolonged delay in reaching the canopy reduced both individual survival and the size of the stem produced by surviving plants the following year. Of the 89 control stems, two (2.3%) produced no aerial stem in April 2001. In contrast, 12 of 95 stems (12.6%) experimentally confined to the understory during the first 3 months of the 2000 growing season had not produced an aerial stem by April 2001. Individual mortality was significantly higher in experimentally confined plants (Fisher's exact test, two‐sided, *p *= .04). Overall, from 1 year to the next, all the stems that died (*N* = 14; mean ± *SD* at *T*_0_ = 3.56 ± 1.59 mm) had a significantly smaller diameter than the ones that survived (*N* = 170; 5.03 ± 2.12 mm) (Mann–Whitney test, *p *= .005).

**Figure 6:**
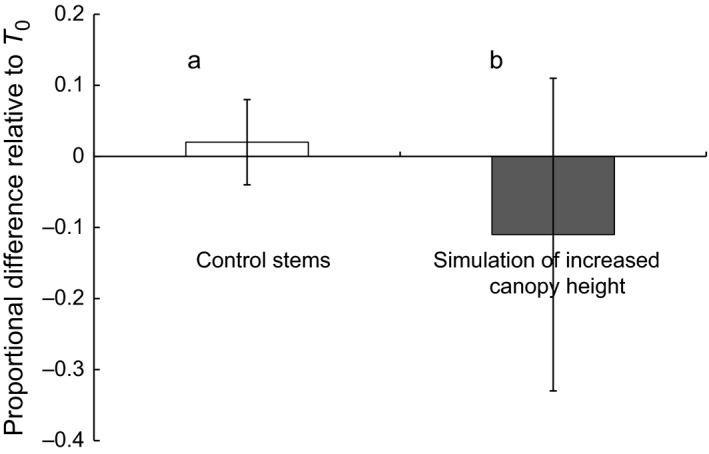
Proportional difference in stem cross‐sectional area from year 2000–2001 between intact stems of control plants (*N* = 87, white bar) and plants having lost 4 months of growth (*N* = 83, black bar). Data are mean ± *SD* of [(*X*_2000_ − X_2001_)/*X*_2000_]. The difference between control and experimental plants was highly significant (*p *= 9.10^−6^, Tukey–Kramer test). This statistical difference is indicated by the letters a and b

Comparison of the cross‐sectional area of stems produced by the same plant in 2000 and in 2001 showed a highly significant effect of treatment (*F*_1, 168_ = 31.10, *p *< .0000001). In the control group, stem cross‐sectional area was not significantly different across the 2 years. In contrast, the cross‐sectional area of experimental stems was reduced on average by 12% (from 9.6 ± 2.9 mm² in 2000 to 8.4 ± 3.1 mm² in 2001) (Figure 6). This reduction in stem cross‐sectional area was likely due to both the greater material cost of producing the previous year's stem and to the considerable reduction of the period available for photosynthesis and thus in the quantity of tuber reserves stored for the next cycle.

### 3.3 Effect of stem size on the timing of stem emergence and the end of the leafless phase

Some stems emerged above ground level well before the beginning of the short rainy season. At the first census date (12 February), 133 of 275 stems (48.4%) had already emerged (cohort 1). A further 59 stems (cohort 2, 21.4% of the total) had emerged by 8 March, 47 more stems (cohort 3, 17.1%) by 12 April and 36 more (cohort 4, 13.1%) by 24 April. Timing of emergence depended on stem cross‐sectional area; larger stems emerged significantly earlier. Stems of cohort 1 had cross‐sectional areas of 35.3 ± 13.5 mm^2^ (mean ± *SD*; range 7.5–75.4 mm^2^), and those of cohorts 2 (32.0 ± 12.7 mm^2^, range 12.6–70.3), 3 (29.5 ± 10.3 mm^2^, range 11.4–56.7), and 4 (21.5 ± 10.9 mm^2^, range 7.5–53.6) were successively smaller (Kruskal–Wallis test, *H*_3, 275_ = 32.28, *p* < .0001). Pairwise comparisons between cohorts are presented in Table 2. The strong correlation between cross‐sectional area of stems and size of the tubers that produce them makes it likely that early‐emerging stems came from plants with larger tubers.

**Table 2:** Differences in cross‐sectional area between stems that emerged in each of the four census intervals

	12 February	8 March	12 April	24 April
12 February	**_**	*Z* = 1.71	***Z*** **=** **2.67**	***Z*** **= 5.23**
		*p* = .09	***p*** **=** **.008**	***p*** **< 10^−6^**
8 March		**_**	*Z* = 0.72	***Z*** ** = 3.83**
			*p *= .47	***p *** **= .0001**
12 April			**_**	***Z*** ** = 3.45**
				***p *** **= .0005**

Significant results are in bold.

In contrast, timing of the end of the leafless phase was negatively correlated with stem cross‐sectional area. The 22 stems that had already produced foliage leaves by the 24 April census all had small stem cross‐sectional areas (mean ± *SD* 16.0 ± 8.0 mm^2^). In comparison, the 253 stems that had not produced any foliage by this final census had larger stem cross‐sectional areas (33.1 ± 12.8 mm^2^). This difference was highly significant (Mann–Whitney, *Z* = 5.85, *p* < .000001). We observed that these early‐emerging crowns of small‐stemmed plants were all located in the shaded understory at heights below 6 m. Over several years of study, this was the first time we observed *D. praehensilis* individuals developing their foliar crown in suboptimal conditions. Stem size (and tuber size) thus appeared to affect the plant's ability to reach the favorable light conditions of the canopy.

## 4 DISCUSSION

Ant‐exclusion experiments confirmed the crucial role of opportunistic ants (Di Giusto et al., 2001) in limiting the presence of the larvae of *L. latipennis*. Because of the plant's high vulnerability during the leafless growth phase, real and simulated herbivory that removed small amounts of tissue imposed disproportionate costs to the plant. Plants subjected to natural or simulated herbivory had to reiterate an establishment growth and saw their exploratory growth rates reduced by up to a third, resulting in a delay in reaching the canopy. Furthermore, attacks on short‐height stems increased the probability of further herbivory, and so further lengthened the amount of time required to reach the canopy. Finally, the experiment of prolonged stem growth showed a major cost in terms of lost opportunities. A shortened productive period likely reduced the replenishment of tuber reserves, and thus the probability of survival and growth abilities of surviving individuals in the following year. Our study also suggests that the strategy of *D. praehensilis* to prevent and respond to its specialist herbivore consists in maximizing stem growth speed and precocity through an over‐accumulation of underground reserves.

### 4.1 Short‐term costs

The most vulnerable point in *D. praehensilis**’*** cycle is the high cost of herbivory during the annual trip to the canopy. This high cost results from its growth strategy, which is conditioned by the constraints imposed by its physical environment, by the presence of a specialist herbivore, by the vine life form, and by the monocot habit. First, in monocots, the limited number of meristems (Holttum, 1955) restricts the possibility of ramification. Furthermore, because of the greater complexity of their vascularization, “vascular development of a Monocots’ branch often takes place over a longer period of time” (Zimmermann & Tomlinson, 1972). Second, the annuality of the stem and the need to reach maximal solar exposure before branching results in a strong dominance of the apical meristem. This precludes the possibility of modifying the plant architecture through the activation of latent axillary meristems following herbivory (Marquis, 1996; Wise & Abrahamson, 2008). Third, both the ecological and architectural constraints of vines and monocotyledons necessitate an establishment growth stage for each new ramification. On the one hand, most monocotyledons require thickening of the meristem to the primary diameter that will be maintained throughout the lifespan of the shoot system (Holttum, 1955). On the other hand, vines need a solid base to reach surrounding plant supports.

These architectural and ecological constraints tend to shape both resistance (the ability to prevent or lessen the herbivore damage) and tolerance (the ability to maximize recovery following herbivory) (Crawley, 1989; Núñez‐Farfán, Fornoni, & Valverde, 2007). Although tolerance is likely an effective strategy against specialist herbivores that are relatively immune to the plant's chemical defenses (Strauss & Agrawal, 1999), *D. praehensilis* showed no overcompensation after herbivory but rather a reduced rate of exploratory growth. This reduction of the growth rate is both potentially the most important and the one for which our estimates are most subject to uncertainty. However, two findings suggest a lasting reduction in growth rates. First, by the end of the 20‐day experiment, the maximal rates of extension growth of damaged stems were still reduced by about one‐third compared to those of intact stems. Second, the slope of the growth rates of damaged stems appeared to cease increasing 4 days before we terminated observations (Figure 4a). Further observations are needed to determine why the reduction in growth rate persists. We hypothesize an imperfect vascularization between the main stem and its ramification due to the absence of secondary cambium in Monocots (Tomlinson, 1980) or an effect of the bigger diameter of the ramification that might compromise the growth in length.

**Figure 7:**
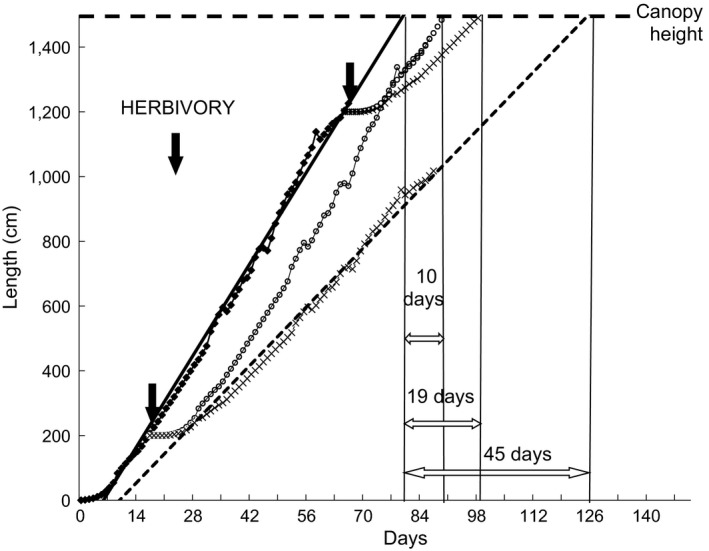
Modeling of growth to the canopy by stems of *Dioscorea praehensilis* with and without herbivory. Using the data from the 20‐day experiment, we estimated by extrapolation the effect of herbivory on the total time lost in reaching the canopy. Assuming a canopy height of 15 m above ground, we took into account the sources of delay we measured: time required to activate lateral buds (4 days), time required to complete the new phase of establishment growth (8 days), and a lasting reduction of up to one‐third of the maximal growth rate even 20 days later, during exploratory growth. The major uncertainty was the extent to which there was a long‐lasting reduction of growth rates. Depending on the type of analysis conducted, the extension growth rates of cut plants had either attained a slope comparable to that for intact stems by the end of the experiment (Figure 6b) or a slope that was still up to one‐third lower than that for intact plants (Figure 6a). We, thus, examined both alternatives. The curve with black squares shows the growth of intact stems from ground to canopy (the black line represents the tangent of the curve). We compared the effects of herbivory on the apex at 200 and at 1,200 cm height and calculated the growth of the ramification. Curves drawn with white circles show the growth of the new ramification with a rate of exploratory growth equivalent to that of intact stems after the activation of its lateral buds and a new establishment growth. Curves drawn with black crosses are similar to the previous curves, but with a rate of exploratory growth reduced by 30% relative to that of intact stems (the dashed line represents the tangent of the curve). Calculation of time lost in reaching the canopy is based on extrapolation from regression lines of growth rate of damaged and intact stems

A simulation of growth (Figure 7) using our previous results led to a total delay of 10 days in reaching the canopy for equal rates of exploratory growth before and after herbivory. In contrast, for a 30% reduction of rates of exploratory growth, the delay in reaching the canopy varied from 21 to 45 days depending on the height of the apex when herbivory occurred (Figure 7). The earlier the attack, the greater the cost imposed. As found in earlier studies (Marquis, 1992b; Marquis et al., 1997; Strauss & Agrawal, 1999; Tiffin, 2002), the impact of herbivory in *D. praehensilis* depended on the timing of the attack within the plant's biological cycle. A delay of 45 days in reaching the canopy represents a reduction of up to 25% of the entire productive season for the plant's foliar crown. The estimated loss of photosynthetic production confirms the disproportionate cost of herbivory relative to the amount of biomass consumed.

Our study is also consistent with others (Blundell & Peart, 2001; Fornoni, 2011; Wise & Abrahamson, 2008) in finding that tolerance responses depend not only on plant architecture but also on environmental context (e.g. limiting light, water, and nutrients). A combination of constraints and limiting factors might explain why the plant is unable to overcompensate despite the high cost entailed by failing to reach the canopy in time. For example, Hawkes and Sullivan (2001) noticed in their review on plant responses to herbivory in different resource conditions that, among the studied plant species, the only plants able to truly overcompensate were grass‐like monocotyledons with basal meristems growing in high resource environments. Plants with simple architecture and strong constraints on their growth strategies may offer valuable insights into how growth strategies and architecture affect the tolerance of plants to herbivory and shape the evolution of defenses (Tomlinson, 1980).

Several authors have discussed how much simulated herbivory tells us about the effects of real herbivory (Tiffin, 2000; Tiffin & Inouye, 2000). In our study, the time required to activate an axillary bud was significantly different between real and simulated herbivory. This is likely due to the gradual destruction of the apex of the stem by larvae in real herbivory (in contrast to an instantaneous event for simulated herbivory), which appeared to induce an early release of lateral buds from apical dominance, even before our assessment that the apex was destroyed.

### 4.2 Long‐term costs

As Koricheva (2002) suggests, “The prevalence of costs in the field and their persistence (…) both imply that fitness costs may arise not only due to trade‐offs in the allocation of common limited resources between defense, growth and reproduction within an individual plant, but also through interactions between plants and external factors in their environment.” Decreased production by the aerial apparatus of *D. praehensilis* due to delay, to deployment in less favorable environments, or both, reduces plant performance (survivorship and size) in the following year. A smaller stem diameter in the year following the prolonged growth in the understory likely indicates that growth rate to the canopy and photosynthetic surface will also be reduced, resulting in a long‐term reduction in plant fitness. The cost was higher for the smallest individuals of *D. praehensilis*, a result consistent with the findings of Boege and Marquis (2005) that the costs of herbivory vary over the plant's ontogeny. We propose that severe herbivore attack, through a reduction either in the length of the growing season or in the quality of the light environment attained at the end of extension growth, could lead to costs that persist, or even increase, over many seasons. Plants may be expected to maintain tuber restocking as fully as possible, perhaps at the cost of reduced investment in flowering and fruiting in the current season.

Faced with a specialist herbivore able to subvert the plant's chemical defenses and sequester them in its own defense (Di Giusto et al., 2001), an increase in the plant's chemical defenses might be counterproductive by leading to increased effectiveness of the beetles’ fecal shields against ants (Baldwin & Preston, 1999). Under these circumstances, the most effective defense may be to amass a larger tuber, enabling the plant to grow earlier and faster and providing the plant with insurance against severe levels of herbivory.

**Figure 8:**
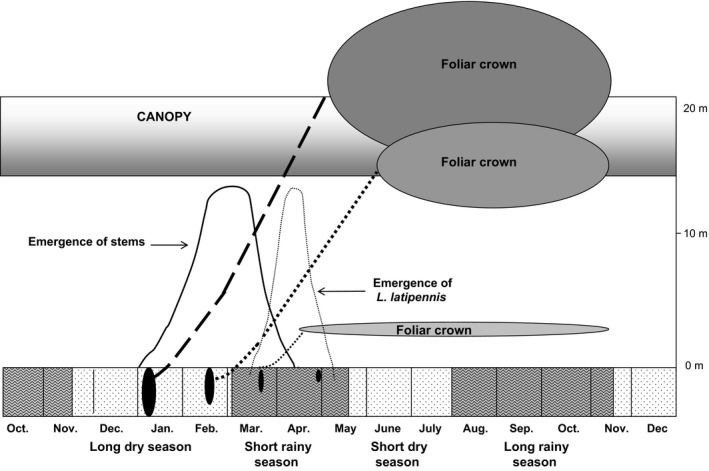
How tuber size affects the phenology of growth to the canopy and establishment of the foliar crown, in the growth strategy of *Dioscorea praehensilis*. Patterns shown are those observed when the apex is not attacked by herbivores during transit to the canopy. Timing of stem emergence, growth rates and timing of the end of the leafless phase for plants of varying stem and tuber size are based on data presented in this study. Depictions of the relative size of foliar crowns and their height in relation to the canopy are based on our unpublished observations

Our results support our hypothesis that the plant's aerial apparatus and underground reserves are tied together in a cycle of reciprocal annual renewal. First, the greater the quantity of reserves stored in the tuber, the larger the cross‐sectional area of the stem produced. Larger stems grew more rapidly in length (Figure 5) and emerged from the tuber earlier in the middle of the long dry season (Table 2, Figure 8). Thus, storage of a large amount of underground reserves allows the plant not only to put in place a larger photosynthetic surface, but also to ensure that it rapidly reaches the favorable light conditions of the canopy (Figure 8). Early emergence and fast growth convey at least two advantages (Figure 8): (1) Crowns of early‐emerging plants have already reached the canopy near the end of the short rainy season and thus benefit from favorable light conditions throughout the season; and (2) stem apices of early‐emerging plants traverse the understory before the peak emergence of *L. latipennis*. This precocity in phenology allows them to partly escape the plant's principal herbivore or at least suffer less damage if herbivory does occur. In view of these advantages of early emergence, why do smaller plants emerge later? Greater water reserves may enable larger plants to initiate growth well before the end of the dry season, while smaller plants (and *L. latipennis* imagos) might have to await the onset of the rains. Water accounts for 70%–75% of the fresh weight of tubers; plants with larger tubers have more of this resource, as well as more energy. They may also have deeper and more extensive root systems, enabling them to tap water reserves deeper in the soil than can smaller plants.

Missed opportunities may drive the plant into a downward spiral. As in other heliophiles, its seedlings establish only in light gaps. But unlike other heliophiles, which maintain their place in the canopy by growing up continuously as the gap closes, this geophyte must make a longer trip from the ground to the canopy with each successive year until the gap closes (McKey et al., 1998). Thus, a steady increase in the rate of growth from year to year is a necessity, given the regeneration niche of *D. praehensilis*. Plants that fail to reach the canopy in time for a full season of photosynthetic activity are more likely to fail to accumulate sufficient reserves to minimize the effects of herbivory and reach the canopy in the following year – by which time the canopy will be higher. Despite several reviews showing an impact of herbivory on growth, flowering, fruiting, or seeding within the same growth season (Strauss & Agrawal, 1999; Wise & Abrahamson, 2008), our study is the first that we are aware of that documents costs persisting *across growth seasons*.

However, annual renewal of the entire aerial apparatus is not the only solution to the problems of viny monocots. Some wild yams closely related to *D. praehensilis*, and sympatric with this species in forests of central Africa, possess aerial systems that last two or more years (Hladik et al., 1984). These species, exemplified by *D. mangenotiana*, have very large stems, enormous tubers (up to 200 kg), and longer‐lived, much tougher leaves. They are found in mature forests with very high canopies (up to 50 m). Among forest and savannah‐dwelling species of *Dioscorea*, there is thus scope for comparative studies to test predictions about the ecophysiological diversity in storage organs and aerial apparatus that should accompany this diversity in life cycles.

## 5 CONCLUSION

*Dioscorea praehensilis* has evolved a strategy, shaped by the ecological and architectural constraints it faces, that centers on the annual and reciprocal renewal of both the aerial apparatus and underground tuber. The plant's aerial apparatus is highly vulnerable to herbivory occurring during the annual trip to the canopy. Herbivore attacks that destroy the apical meristem greatly reduce stem growth rate to the canopy. By temporarily halting growth, herbivory reduces the advantage to the plant of having a large amount of stored reserves. Although herbivory removes little material, by delaying growth to the canopy it substantially reduces the period of photosynthetic activity and likely weakens future chances for the plant to complete its biological cycle. This cost of lost opportunities appears to represent the most significant effect of herbivory on plant fitness. Future studies comparing ecological adaptations and life cycles of *Dioscorea* species living in open savannah environments, low‐stature secondary forests, and mature forests varying in canopy height could provide new insights into the role of herbivory in the evolution of resource storage strategies.

## CONFLICT OF INTEREST

None declared.
