# Supplementary material for: Facing herbivory on the climb up: Lost opportunities as the main cost of herbivory in the wild yam Dioscorea praehensilis
Source: Ecol Evol. 2017 Jul 11;7(16):6493–506. doi: 10.1002/ece3.3066 (PMC5574755; doi:10.1002/ece3.3066)
Supplement: Supplementary file 1 [file ECE3-7-6493-s001.docx]

**Supporting information**

**Stem cross-sectional area as an estimator of the quantity of tuber reserves**

**Material and Methods**

The quantity of reserves stored in the tuber is a key variable, as it is likely to affect both the rate at which the stem can grow to the canopy, and the size of the photosynthetic surface put in place at the end of this growth. However, quantity of tuber reserves can only be determined by destructive sampling. In March 1999, when stems had just begun their growth toward the canopy, we excavated 49 tubers of *D. praehensilis* to determine their fresh mass. We also measured the diameter (at 1 m height) of the aerial stem produced by each tuber, to determine whether the stem diameter could be used as an indicator of the mass of stored reserves. The relationship between stem cross-sectional areas and tuber mass was analysed by a regression using a log-linear model. The slope of the regression line was compared to isometry using a *t*-test.

**Results**

The fresh mass of 49 tubers of individuals of *D. praehensilis* chosen to represent a broad range of size as judged by stem cross-sectional area varied from 0.05 to 9.75 kg (mean + s.d. = 2.20 + 2.64 kg). The cross-sectional surface of stems significantly increased with the fresh mass of the tubers (supplementary data, Fig. 6). Stem diameter (and cross-sectional surface) can thus be used as an indicator of the amount of tuber reserves available for growth of the aerial apparatus of the plant. The slope of the regression of tuber mass on stem cross-sectional area is very close to 1 (y = 1.09x - 1.35), but the relationship is not isometric [*t* (47) = -7.19, *P* < 10^-6^]; an increase in tuber mass is accompanied by a slightly smaller increase in stem cross-sectional area.

**Fig. 5:** Correlation between cross-sectional area of stem and fresh mass of tuber of *D. praehensilis* (N = 49 tubers and stems). Data were log-transformed. Cross-sectional area (in mm^2^ (A = πR²)) of the stem was significantly correlated with the fresh mass (in kg) of the tuber (r^2^ = 0.55, *P* < 10^-6^). The solid line is the regression (log linear model) of tuber mass on stem cross-sectional area (y = 1.09x – 1.35).
